# Impact of Two Surgical Techniques for Umbilical Hernia Repair, With and Without Peritoneal Opening, on Pain Response, Sedation, and Oxidative Stress in Calves

**DOI:** 10.3390/vetsci12100963

**Published:** 2025-10-09

**Authors:** Claudia Interlandi, Filippo Spadola, Fabio Bruno, Giuseppe Bruschetta, Francesco Macrì, Andrea Spadaro, Giovanni Barone, Giovanna Lucrezia Costa

**Affiliations:** 1 Department of Veterinary Sciences, University of Messina, 98168 Messina, Italy; cinterlandi@unime.it (C.I.); filippo.spadola@unime.it (F.S.); giuseppe.bruschetta@unime.it (G.B.); fmacri@unime.it (F.M.); andrea.spadaro@unime.it (A.S.); glcosta@unime.it (G.L.C.); 2 Freelance Veterinarian, 97015 Modica, Italy; giobarone96@gmail.com

**Keywords:** analgesia, inflammatory biomarkers, herniorrhaphy, bovine surgery

## Abstract

**Simple Summary:**

This study presents a comparative evaluation of two surgical techniques for umbilical hernia repair in calves, focusing on their effects on homeostasis, sedation quality, pain response, and oxidative stress. By measuring key physiological parameters, pain scores, and biomarkers such as malondialdehyde (MDA) and serotonin (5-HT), this study provides a comprehensive analysis of the systemic impact of surgical invasiveness. These findings offer relevant insights for veterinary practitioners and researchers aiming to refine surgical approaches to enhance animal welfare and reduce postoperative complications. Moreover, this study’s methodological rigor and clinical relevance contribute to the growing body of knowledge on minimally invasive techniques in large animal surgery, supporting evidence-based decision-making.

**Abstract:**

This study aimed to compare effects on homeostasis and postoperative outcomes of two surgical techniques for umbilical hernia repair in calves. Fifty-two calves were enrolled and randomly assigned to two groups: Group A (open technique) and Group C (closed technique). This was a prospective controlled clinical trial. Sedation was induced with romifidine, and butorphanol. Cardiopulmonary parameters, sedation scores, and body temperature were recorded at multiple perioperative timepoints (T0–T8). Postoperative pain was assessed using the UNESP-Botucatu UCPS-IV scale. Oxidative stress was evaluated by measuring serum malondialdehyde (MDA) and plasma serotonin (5-HT) concentrations at T0 and 36 h postoperatively T9. Physiological parameters remained within normal limits in both groups. Postoperative pain scores were significantly lower in Group C than in Group A (*p* < 0.001), with later onset of rescue analgesia 40 vs. 30 min post-standing, respectively, *p* < 0.001. MDA levels increased postoperatively in both groups, with a greater rise in Group A (*p* < 0.001), 5-HT decreased in Group A and increased in Group C (*p* = 0.020). The closed surgical technique for umbilical hernia repair, avoiding peritoneal opening, was associated with reduced postoperative pain and oxidative stress, suggesting it is a less invasive than the open surgical technique.

## 1. Introduction

Umbilical hernia in neonatal calves is characterized by the protrusion of abdominal contents through an incompletely closed umbilical ring, which is formed by the remnants of fetal structures such as the urachus and umbilical vessels [1,2,3]. Small hernias typically contain reducible viscera, including the small intestine (enterocoele) or omentum (epiplocele), whereas larger defects can lead to complications such as adhesions, edema, and tissue necrosis. Surgical repair remains the definitive treatment, primarily performed by either an open technique involving peritoneal entry or a closed approach without peritoneal breach [4,5,6]. Both surgical methods, however, induce tissue injury and may provoke postoperative oxidative stress and inflammation, measurable by biomarkers including malondialdehyde (MDA) and serotonin (5-hydroxytryptamine, 5-HT) [7,8,9,10,11]. These biomarkers reflect cellular oxidative damage and neuromodulatory responses, respectively, and can provide insight into the physiological impact of surgical interventions. Despite the clinical relevance of umbilical hernia repair in calves, comparative data on the systemic physiological and biochemical consequences of the open versus closed surgical techniques are limited. In particular, little is known about how these approaches differ in terms of nociceptive stimulation, sedation depth, and oxidative stress responses under standardized anesthesia and analgesia protocols. Given that serotonin plays a multifaceted role in both central and peripheral physiological processes, including modulation of pain, inflammation, and gastrointestinal function [12,13,14], and that oxidative stress from reactive oxygen species (ROS) can exacerbate tissue damage and affect neurotransmitter availability [15,16,17,18,19,20,21,22], investigating these markers in the surgical context is crucial. Understanding these differences may aid in refining surgical and anesthetic management to optimize animal welfare and recovery. The primary objective of this study was to evaluate and compare the physiological and biochemical responses associated with open (with peritoneal opening) versus closed (without peritoneal opening) umbilical hernia repair techniques in calves. We hypothesized that the closed technique would result in reduced nociceptive input, lower oxidative stress, and more stable sedation profiles compared to the open approach. Specifically, we assessed postoperative pain scores, sedation depth, and serum MDA and plasma serotonin concentrations as indicators of surgical invasiveness and homeostatic disruption. This work aims to provide evidence-based guidance to improve surgical outcomes and minimize postoperative complications in veterinary practice.

## 2. Materials and Methods

This prospective, randomized clinical trial was conducted in accordance with Italian Legislative Decree No. 26 of 4 March 2014 and approved by the Institutional Animal Care and Use Committee (IACUC) of the University of Messina (Protocol No. 031/2019 dated 23 June 2019). All procedures adhered to national (Italian D.M. 116/1992) and international guidelines (EU Directive 2010/63/EU; U.S. Public Health Service Policy on Humane Care and Use of Laboratory Animals). Informed written consent was obtained from all animal owners prior to enrollment. Sample size was calculated using G*Power 3.1 software. An a priori ANOVA (fixed-effects, omnibus, one-way) was performed with an effect size (f) of 0.40, alpha level of 0.05, power of 0.80, and two treatment groups. Outcome variables included differences in pain response, sedation depth, and oxidative stress. A total of 59 calves (14 males, 45 females; Friesian, Alpine Brown, Modicana, and crossbreeds) presented for surgical umbilical hernia repair between 2021 and 2024 during spring and autumn. Only animals classified as ASA Physical Status II [23], based on physical examination, were eligible. Seven calves were excluded due to omphalophlebitis or other concurrent disease, resulting in a final sample size of 52 (11 males, 41 females). Inclusion criteria were uncomplicated, reducible umbilical hernia measuring 5–13 cm on the longest axis. Calves were randomly assigned (simple randomization by draw) to one of two treatment groups: Group A (*n* = 26; open herniorrhaphy with peritoneal incision) or Group C (*n* = 26; closed herniorrhaphy without peritoneal incision). Food was withheld for 6 h and water for 1 h prior to surgery. All procedures were performed on-farm. Body weight was determined using an OCS300 Zoo Piro scale (Cruto, Calabria, Italy). After a 50-min acclimatization period, sedation was induced with intramuscular romifidine (0.08 mg/kg; Sedivet^®^, Boehringer Ingelheim, Milan, Italy). Following adequate sedation, a 14G × 5” intravenous catheter was placed in the jugular vein, and lactated Ringer’s solution was administered at 10 mL/kg/h. Butorphanol (0.02 mg/kg; Dolorex^®^, MSD Animal Health, Milan, Italy) was diluted with 0.9% saline to 40 mL/syringe and administered locally (infiltration of skin and muscle) and intraperitoneally within the hernial sac in both groups. Physiological parameters were monitored using a multiparameter device (EDAN Instruments, Naples, Italy). Heart rate (HR, bpm) and SpO_2_ (%) were measured via lingual pulse oximetry; blood pressure (SAP, MAP, DAP; mmHg) was assessed by the oscillometric method using a tail cuff (30–40% tail circumference). Respiratory rate (RR) was measured by thoracic excursion, and body temperature (T, °C) with a digital veterinary thermometer (GIMA). Measurements were recorded at the following time points: T0 (baseline), T1 (15 min post-sedation), T2 (5 min after local anesthesia), T3 (skin incision), T4 (hernial sac exposure), T5 (hernial sac isolation), T6 (hernia reduction), T7 (muscle suturing), and T8 (skin suturing). Intraoperative time was recorded from T3 to T8. Rescue analgesia (2% lidocaine 2 mg/kg) was administered if HR, RR, or SAP increased >20% from T1.

### 2.1. Sedation Assessment

Sedation was scored (0–3) by three blinded observers using a descriptive scale:

0—Awake;
1—Mild sedation (easily handled, poor muscle relaxation);
2—Moderate sedation (easily handled, moderate relaxation);
3—Deep sedation (easily handled, good relaxation).

Scores were recorded at each time point (T0–T8).

### 2.2. Surgical Techniques

The animals were sedated and positioned in dorsal recumbency to facilitate access to the umbilical area. Fifteen minutes prior to surgery, analgesic infiltration was performed at the incision site using butorphanol. The surgical site was prepared by shaving the area (trichotomy) and disinfecting with an alcohol and iodine solution (at least four passes were performed). In Group A (Open Method), an elliptical incision was made at the skin level over the swelling corresponding to the hernial sac. The hernial sac was then isolated from the surrounding tissues, either manually or with the aid of scissors. Once exposed, the sac was incised through its full thickness, and the hernial ring along with the contents (intestine, omentum, or adipose tissue) were carefully evaluated. Any adhesions between the viscera and the hernial sac were removed using blunt dissection or manual tearing. The margins of the hernial ring were scarified. Suturing was performed using a horizontal mattress technique with absorbable polyglactin 910 sutures (size 1 or 2, depending on the size of the animal). The hernial sac was then shaped to form an autologous vascular flap, which reinforced the herniorrhaphy. The subcutaneous layer was closed with “X” or “8” sutures, and the skin was sutured with interrupted knots. In Group C (Closed Method), after skin incision and dissection of the surrounding tissues, the hernia contents were manually reduced into the abdominal cavity without making an incision in the hernial sac. The outer perimeter of the hernial ring was then debrided and sutured from the outside using a series of vertical interrupted sutures, employing polyglactin 910 sutures (size 2–3). Suturing of the overlying layers was performed as in the previous method.

### 2.3. Postoperative Pain Assessment

Pain was assessed using the UNESP-Botucatu Unidimensional Composite Pain Scale (UCPS-IV) by three blinded observers at 10-min intervals (M1–M6) after the calf returned to standing. The scale included five behavioral domains: locomotion, interaction, activity, appetite, and attention to the surgical site [24,25]. Each domain was scored 0–2; total pain score ranged from 0 (no pain) to 10 (severe pain). A score ≥ 4 triggered rescue analgesia (flunixin meglumine 2.2 mg/kg IV; Finadyne^®^, Schering-Plough, Kenilworth, NJ, USA). After the pain scale assessment, all calves were given the same daily dose of flunixin meglumine for three days after surgery.

### 2.4. Sample Collection

Blood samples (20 mL) were collected from the jugular vein at baseline (T0) and 36 h post-surgery (T9) by the same operator. Samples were divided into serum (VACUETTE^®^ Serum Z, Greiner Bio-One, Kremsmünster, Austria) and EDTA (K3) tubes (Greiner Bio-One, Kremsmünster, Austria), stored at 4 °C, and centrifuged at 2000 g for 10 min at 4 °C. Plasma and serum were separated and stored until analysis. Laboratory staff were blinded to treatment allocation. Plasma 5-HT levels were quantified using a validated ELISA kit for bovine species (BioVision, Milpitas, CA, USA), following the manufacturer’s protocol. Samples (50 μL) were processed with biotin-detection antibody, SABC, and TMB substrate. Absorbance was measured at 450 nm (reference 630 nm) using a spectrophotometer (A560, Fulltech, Rome, Italy). Assay sensitivity was 1 ng/mL; intra- and inter-assay CVs were 3.8% and 7.7%, respectively [26]. MDA concentrations were assessed by thiobarbituric acid reactive substances (TBARS) assay. TBA (0.11 mol/L) was prepared in NaOH and reacted with samples at 90 °C, followed by cooling. Standards were generated using TMP-derived MDA stock solutions. After derivatization, absorbance was read at 535 nm and 572 nm (BIORAD 680, Milan, Italy), and MDA equivalents were calculated from standard curves [27,28,29].

### 2.5. Statistical Analysis

Data were analyzed using SPSS v27.1 (IBM, Milan, Italy). For repeated measures, linear mixed-effects models were applied with the individual calf as a random effect to account for within-subject correlation over time. Fixed effects included group, time, and their interaction. Initially, an unstructured covariance structure was specified; alternative structures (com-pound symmetry and autoregressive AR (1) were compared using Akaike’s Information Criterion (AIC) to select the best-fitting model. Post hoc comparisons of estimated marginal means across time points were adjusted using the Bonferroni correction to control the family-wise error rate. Where the Shapiro–Wilk test indicated non-normal distributions, equivalent non-parametric approaches were adopted (Mann–Whitney U test for independent samples; Wilcoxon signed-rank test for paired samples). Derived variables, such as percentage changes in MDA and 5-HT between T0 and T9, were analyzed following log10 transformation when required to improve normality. Inter-observer agreement for sedation and pain scores was evaluated using Kendall’s W coefficient. Statistical significance was set at *p* < 0.05 after correction for multiple comparisons.

## 3. Results

There were no significant differences between groups in age, body weight, body condition score, or ASA physical status classification. A total of 26 calves per group were required to detect statistically significant differences with an effective power of 0.80. The interobserver agreement was high (W = 1) (Table 1).

The time required for intraoperative procedures, measured from skin incision (T3) to final suture (T8), differed significantly between groups (*p* < 0.001). Group A had a median duration of 55 min (range: 41–63; mean ± SD: 55.1 ± 6), whereas Group C had a median duration of 43 min (range: 37–56; mean ± SD: 44.1 ± 5) Furthermore, the median recovery time from the quadrupedal position was faster in Group C (110 min, range: 100–120, mean ± SD: 110 ± 8) than in Group A (144 min, range: 135–155, mean ± SD: 145 ± 7) (Table 2).

Heart rate decreased significantly over time in both groups. In Group C, HR was reduced at all time points; in Group A, reductions were observed at selected time points. Between-group comparisons revealed significantly lower HR values in Group C compared to Group A at T3 (*p* < 0.05) and T4 (*p* < 0.01), with no significant differences at T6 or T7 (*p* > 0.05) (Table 3). Group C exhibited significantly lower RR values at T3, T7, and T8 compared to T0 (*p* < 0.001). No significant changes in RR were observed in Group A. No significant differences were found between groups at any time point. In Group A, systolic arterial pressure (SAP) and mean arterial pressure (MAP) significantly decreased from T3 to T8 (*p* < 0.001), while diastolic arterial pressure (DAP) decreased from T2 to T8 (*p* < 0.001). In Group C, SAP decreased from T4 to T8 (*p* < 0.001), MAP from T3 to T8 (*p* < 0.001), and DAP from T1 to T8 (*p* < 0.001), with significance at T1 (*p* < 0.05). Between-group comparisons showed significant differences at SAP T5 (*p* < 0.05), MAP T3 (*p* < 0.001), and DAP T3 and T6 (*p* < 0.001). SpO_2_ values remained stable around 97% in both groups and did not differ significantly at any time point. Body temperature decreased by approximately 1 °C in all calves during anesthesia, with no significant differences between groups. No additional intraoperative analgesia was required in response to noxious stimuli in either group (Table 3).

Sedation scores increased significantly over time in both groups compared to baseline (T0, *p* < 0.001). Between-group analysis showed that scores were significantly higher in Group C from T5 to T8 (*p* < 0.001) (Table 4). All recoveries were uneventful, and no surgical complications were reported.

UNESP-Botucatu pain scores differed significantly between groups from 20 min postoperatively (*p* < 0.001), with lower scores in Group C. In Group A, pain scores varied significantly over time from 20 to 60 min (*p* < 0.001). In this group, eight calves required rescue analgesia at 30 min, three at 40 min, three at 50 min and three at 60 min. In Group C, however, five calves received rescue analgesia at 50 min and four at 60 min (*p* < 0.001) (Table 5). Flunixin meglumine (2.2 mg/kg IV; Finadyne, Schering-Plough Animal Health, Oss, The Netherlands) was administered for the analgesic rescue.

At baseline (T0), serum 5-HT concentrations were significantly lower in Group A (6.7 ± 0.5 ng/mL) compared to Group C (8.6 ± 0.5 ng/mL; *p* < 0.001). At 36 h postoperatively (T9), Group A showed a significant intragroup decrease in 5-HT levels (5.0 ± 0.9 ng/mL; *p* < 0.001), corresponding to a 25.4% ± 14.6% reduction. Group C showed a significant increase in 5-HT at T9 (10.0 ± 2.5 ng/mL; *p* = 0.020), representing a 16.3% ± 29.9% increase from baseline. Between-group differences in 5-HT concentrations remained significant at both time points (*p* < 0.001). At T0, MDA concentrations were significantly lower in Group A (40.5 ± 5.6 nmol/mL) than in Group C (51.9 ± 7.5 nmol/mL; *p* < 0.001). At T9, both groups exhibited significant intragroup increases: Group A reached 81.5 ± 12 nmol/mL (*p* < 0.001), corresponding to a 101.2% ± 40.6% increase; Group C reached 73.4 ± 11 nmol/mL (*p* < 0.001), with a 41.4% ± 29.5% increase. MDA concentrations remained significantly higher in Group A than in Group C at T9 (*p* < 0.001) (Table 6).

## 4. Discussion

This study demonstrates that both surgical techniques for umbilical hernia repair in calves—open (with peritoneal incision) and closed (without peritoneal incision)—result in clinically satisfactory outcomes. However, significant differences were observed between the two approaches in terms of sedation scores, postoperative analgesia, and biomarkers of oxidative and inflammatory stress. The duration of surgery and the median recovery time from the quadrupedal position were both significantly reduced in group C compared to group A. This may promote faster recovery by stabilising body temperature, enabling earlier resumption of feeding and reducing stress [30]. These findings provide new insights into how surgical technique influences physiological stress responses in calves, with implications for postoperative management and animal welfare [31]. Calves undergoing closed hernia repair exhibited significantly lower sedation scores between T5 and T8, suggesting reduced nociceptive stimulation and surgical stress compared to those undergoing open repair. In contrast, the open group required earlier administration of rescue analgesia during recovery, indicating a more intense postoperative pain response. This study selected a cut-off score of ≥4 on the Botucatu one-dimensional composite pain scale for postoperative rescue analgesia a priori. The obtained scores show that several subjects in group A required rescue analgesia after only 20 min of monitoring, while some subjects in group C required it only after 50 min [25,32]. Despite these differences, intraoperative physiological parameters such as heart rate, respiratory rate, and blood pressure remained within baseline ranges in both groups, and no calves required rescue analgesia intraoperatively. These results suggest that the sedation protocol based on romifidine and butorphanol provided effective analgesia during surgery, regardless of the technique employed, consistent with previous reports [2,31,32,33,34,35,36]. Oxidative stress, as indicated by plasma malondialdehyde (MDA) concentrations, increased significantly in both groups postoperatively, with a more pronounced rise in the open surgery group. This aligns with literature describing the role of surgical tissue trauma, ischemia, and reperfusion in promoting lipid peroxidation and oxidative stress [29]. Although studies increasingly emphasize minimizing surgical stress through optimized anaesthetic protocols and techniques, few have quantified oxidative stress using objective biomarkers such as MDA [5,29]. Interestingly, we also observed divergent patterns in plasma serotonin (5-HT) levels between groups. In the closed group, 5-HT concentrations increased significantly postoperatively, while a significant decrease was seen in the open group. Although elevated 5-HT is typically associated with surgical stress, the observed reduction in Group A may reflect an amplified or heightened inflammatory response caused by more extensive tissue manipulation, particularly involving peritoneal structures. These findings support the hypothesis that 5-HT may serve as a context-dependent biomarker of surgical inflammation, as previously suggested in canine studies [27]. However, baseline differences in 5-HT between groups necessitate caution when at-tributing postoperative alterations exclusively to the surgical intervention. While our findings indicate potentially divergent temporal trajectories of 5-HT, we recognize that alternative explanations cannot be ruled out. Variables such as breed-related heterogeneity, sex-dependent differences, or stress induced by handling may have influenced the observed patterns. As these factors could be misleading, it would be inappropriate to interpret fluctuations in 5-HT concentrations as definitive markers of peritoneal inflammation. Consequently, our findings should be considered and reported as hypothesis-generating rather than confirmatory. The interaction between anaesthetic agents and serotonergic pathways may also contribute to the variability in 5-HT responses. Both romifidine (an α2-adrenergic agonist) and butorphanol (a κ-opioid receptor agonist) are known to modulate serotonin dynamics, although their specific effects remain unclear. Previous reports in large animals have shown inconsistent changes in plasma 5-HT under surgical or inflammatory conditions, underscoring the complexity of interpreting this biomarker [27]. Overall, our findings suggest that both increased and decreased plasma serotonin concentrations may reflect different aspects of the inflammatory response, depending on the surgical context. These results warrant further investigation into the utility of 5-HT as a dynamic biomarker of surgical stress. This study has several limitations. First, as a field-based clinical trial, procedures were conducted in a stable environment, which could increase the risk of contamination during open surgeries. Although no clinical cases of peritonitis were observed, the possibility of subclinical peritoneal inflammation cannot be excluded. Second, environmental and climatic conditions may have influenced the effectiveness of the anaesthetic protocol; to mitigate this, all procedures were performed during temperate seasons (spring and autumn), and calves were randomly assigned to treatment groups [37]. Animals were assigned to groups using simple randomization by draw, without stratification based on baseline 5-HT or MDA parameters. Therefore, the observed baseline differences may reflect random variability that was not controlled by the randomization process, particularly given the relatively small group sizes (*n* = 26 per group). However, as this was a field-based clinical study, it was not feasible to process blood samples prior to surgery. Therefore, stratified randomization, which would have al-lowed baseline differences in 5-HT and MDA to be bypassed, could not be performed. Third, although changes in oxidative and serotonergic biomarkers were assessed, other relevant inflammatory mediators (e.g., cytokines, cortisol) were not evaluated, limiting interpretation of the broader immune response. These constraints should be considered when generalizing the results.

## 5. Conclusions

This study provides comparative data on two surgical techniques for umbilical hernia repair in calves, highlighting differences in postoperative stress responses. The closed technique (without peritoneal incision) was associated with lower sedation and pain scores, a shorter time to standing, and less pronounced oxidative and inflammatory responses, as indicated by MDA and 5-HT levels. In contrast, open surgery resulted in an earlier need for postoperative analgesia and greater changes in these biomarkers. These findings support the clinical benefits of a less invasive surgical approach to minimizing postoperative complications and stress. Moreover, this study highlights the potential of combining oxidative stress (MDA) and serotonergic (5-HT) markers for objective assessment of surgical impact. Further studies incorporating additional inflammatory markers are needed to fully elucidate the mechanisms underlying these responses and to refine strategies for perioperative management and welfare optimization in calves.

## Figures and Tables

**Table 1:** Mean and standard deviation of the weight, age, body condition score and ASA status of the subjects enrolled in the two groups.

	Group A	Group C	*p* Value
**Weight (kg)**	76 ± 16	78 ± 18	*p* = 0.39
**Age (Months)**	2.3 ± 1	2.1 ± 0.9	*p* = 0.61
**BCS (Body Condition Score)**	4.1 ± 0.7	4.2 ± 0.7	*p* = 0.10
**ASA (Category)**	2 (1/2)	2 (1/2)	*p* = 0.16

**Table 2:** Time of surgery and recovery of the quadrupedal station in the two monitored groups.

	Surgery Time (min)	Recovery to Standing (min)
**Group A**	55.1 ± 6 ^♣^	145 ± 7 ^♣^
**Group C**	44.1 ± 5	110 ± 8
***p* value**	*p* < 0.001	*p* < 0.001

Values are expressed as mean ± SD. ^♣^ Significant difference between Groups. *p* < 0.05 was considered statistically significant.

**Table 3:** Physiological parameters during umbilical hernia repair in calves treated with (group A; n = 26) and without peritoneal opening (group C; n = 26).

Time		T0	T1	T2	T3	T4	T5	T6	T7	T8
**HR,** **(beats/** **min)**	**Group A**	73 ± 8	68 ± 8 *	69 ± 8 *	72 ± 7 ^♣^	74 ± 6 ^♣^	75 ± 7 *	75 ± 7 *^♣^	74 ± 6 *^♣^	73 ± 7
	**Group C**	71 ± 8	66 ± 9 *	65 ± 7 *	66 ± 8	67 ± 9 *	70 ± 8	68 ± 4 *	68 ± 6	68 ± 8 *
**RR** **(breaths/** **minutes)**	**Group A**	43 ± 6	42 ± 6	42 ± 6	41 ± 7	43 ± 7	45 ± 7	45 ± 8	43 ± 7	42 ± 6
	**Group C**	42 ± 5	41 ± 7	40 ± 8	39 ± 7 *	40 ± 8	42 ± 7	40 ± 7	38 ± 7 *	39 ± 6 *
**SAP** **(mmHg)**	**Group A**	139 ± 11	141 ± 13	140 ± 8	134 ± 8 *	132 ± 9 *	131 ± 11 *^♣^	130 ± 13 *	128 ± 13 *	127 ± 15 *
	**Group C**	135 ± 12	138 ± 12	134 ± 12	130 ± 10	125 ± 12 *	121 ± 14 *	121 ± 13 *	122 ± 15 *	124 ± 15 *
**MAP** **(mmHg)**	**Group A**	115 ± 5	120 ± 5	114 ± 9	92 ± 9 *^♣^	92 ± 8 *	91 ± 12 *	89 ± 13 *	92 ± 12 *	94 ± 13 *
	**Group C**	117 ± 10	119 ± 8	113 ± 8	109 ± 7 *	98 ± 5 *	91 ± 9 *	89 ± 11 *	89 ± 12 *	92 ± 11 *
**DAP** **(mmHg)**	**Group A**	76 ± 3	75 ± 5	69 ± 10 *	67 ± 8 *^♣^	67 ± 9 *	66 ± 12 *	68 ± 10 *^♣^	67 ± 11 *	67 ± 12 *
	**Group C**	73 ± 7	69 ± 9 *	64 ± 8 *	58 ± 11 *	60 ± 9 *	62 ± 9 *	59 ± 8 *	61 ± 7 *	64 ± 8 *
**T°** **(°C)**	**Group A**	39.7 ± 0.6								38.1 ± 0.7 *
	**Group C**	39.5 ± 0.5								38.4 ± 0.6 *

Heart rate (HR, beats/min), respiratory rate (RR, breaths/minutes), body temperature (T°), non-invasive systolic (SAP), mean (MAP), diastolic (DAP) and arterial blood pressure (mmHg) were recorded at different times: at the time of the physical examination (T0; basal values), after sedation with the patient in dorsal recumbency (T1), five minutes after administration of the local anaesthetic (T2), after skin incision (T3), after exposure of the hernia sac (T4), hernia surgery (T5), hernia reduction (T6), muscle planes suturing (T7), skin suturing (T8). * Significant difference from baseline within treatment. ^♣^ Significant difference between Group A and Group C at the corresponding time point; *p* < 0.05 was considered statistically significant.

**Table 4:** Sedation score during umbilical hernia repair in calves treated with and without peritoneal opening (group A, *n* = 26; group C, *n* = 26). Values are expressed as median and range.

Time	T0	T1	T2	T3	T4	T5	T6	T7	T8
**Group A**	0 (0/0)	1 (1/1) *	3 (2/3) *	3 (2/3) *	3 (2/3) *	2 (2/2) *	2 (1/2) *	2 (1/2) *	2 (1/2) *
**Group C**	0 (0/0)	1 (1/1) *	3 (2/3) *	3 (2/3) *	3 (2/3) *	3 (2/3) *^♣^	2 (2/2) *^♣^	2 (2/2) *^♣^	2 (2/2) *^♣^

Descriptive scale used to assess sedation, with scores from 0 to 3: 0—animal awake; 1—mild sedation, the calf is easily manageable but muscle relaxation is poor; 2—moderate sedation, the calf is easily manageable but muscle relaxation is moderate; 3—deep sedation, the calf is easily manageable with good muscle relaxation. basal values (T0), after sedation with the patient in dorsal recumbency (T1), five minutes after administration of the local anaesthetic (T2), after skin incision (T3), after exposure of the hernia sac (T4), hernia surgery (T5), hernia reduction (T6), suturing of the muscle planes (T7), suturing of the skin (T8). * Significant difference from baseline. ^♣^ Significant difference from baseline between groups, *p* < 0.05 was considered significant.

**Table 5:** The UNESP-Botucatu composite one-dimensional pain scale (UNESP-Botucatu UCPS-IV) was used to assess postoperative pain in both groups.

	M1	M2	M3	M4	M5	M6
**Group A**	1 (0/2)	2 (0/3) *^♣^	3 (0/5) *^♣^	3 (2/5) *^♣^	4 (2/5) *^♣^	4 (3/5) *^♣^
**Group C**	1 (0/2)	1 (0/2)	1 (0/2)	1 (0/2) *	2 (0/5) *	3 (1/5) *

The table shows the values at M1 (10 min), M2 (20 min), M3 (30 min), M4 (40 min), M5 (50 min) and M6 (60 min) after returning to a standing position, expressed as median and range. * Significant differences within the group (*p* < 0.001). ^♣^ Significant differences between groups (*p* < 0.001). A *p*-value < 0.05 was considered statistically significant.

**Table 6:** Serotonin and Malondialdehyde (MDA) concentrations in calves treated with (group A; *n* = 26) and without peritoneal opening (group C; *n* = 26). Values are expressed as mean ± SD.

Groups	T0	T9	*p* Value (Intragroup)
**Group A 5-HT**	6.7 ± 0.5	5 ± 0.9 *	*p* < 0.001
**Group C 5-HT**	8.6 ± 0.5 ^♣^	10 ± 2.5 *^♣^	*p* = 0.020
***p* value** (difference between groups)	*p* < 0.001	*p* < 0.001	
**Group A MDA**	40.5 ± 5.6 **^♣^**	81.5 ± 12 *^♣^	*p* < 0.001
**Group C MDA**	51.9 ± 7.5	73.4 ± 11 *	*p* < 0.001
***p* value** (difference between groups)	*p* < 0.001	*p* < 0.001	

The table shows serotonin and MDA levels at baseline (T0) and 36 h after surgery (T9). * Significant difference compared to baseline. ^♣^ Significant difference between groups; *p* < 0.05 was considered significant.

## Data Availability

The original contributions presented in this study are included in the article. Further inquiries can be directed to the corresponding author.

## Abbreviations

The following abbreviations are used in this manuscript:

5-HT	5-Hydroxytryptamine (Serotonin)
MDA	Malondialdehyde
SAP	Systolic Arterial Pressure
MAP	Mean Arterial Pressure
DAP	Diastolic Arterial Pressure
HR	Heart Rate
RR	Respiratory Rate
SpO_2_	Hemoglobin Oxygen Saturation
T0–T9	Time Points (e.g., T0 = baseline, T9 = 36 h post-surgery)
UCPS-IV	Universidade Estadual Paulista—Botucatu, Unesp Composite Pain Scale—Intensive Care Unit Version
